# Research on lower limb lightweight of bionic robot based on lattice structure unit

**DOI:** 10.1038/s41598-025-14679-5

**Published:** 2025-08-11

**Authors:** Huipeng Shen, Liujian Wei, Tianyu Zhang, Xupeng Zhang, Zihao Zheng, Enjiang Han, Shaolong Li

**Affiliations:** 1 Henan Key Laboratory of Superhard Abrasives and Grinding Equipment, Henan University of Technology, Zhengzhou, 450001 China; 2 Engineering Research Center of Integration and Application of Digital Learning Technology, Ministry of Education, Beijing, 100081 China; 3 AECC Hunan Aviation Powerplant Research Institute, Zhuzhou, 412002 China

**Keywords:** Lattice structure, Mechanical performance, Numerical simulation, Experimental testing, Lightweight design of robot lower limb, Mechanical engineering, Design, synthesis and processing

## Abstract

This study presents a lightweight design methodology for the lower limbs of bionic robots based on lattice structural units. Firstly, an innovative structure configuration library is created by applying topology optimization, and then the lattice structure is regularized. A specific stiffness standard has been established for evaluating the mechanical properties of the lattice structure. The mechanical properties of 20 lattice structural units under basic conditions, including compression, bending, and torsion, are analyzed. A new method for calculating weights in composite conditions is introduced to aid in selecting suitable lattice structures for complex scenarios. An experimental setup is constructed to verify the mechanical performance of the lattice structures. The Analytic Hierarchy Process (AHP) is utilized to analyze the loads on individual components and to determine the proportion of each condition in complex scenarios, thereby identifying the optimal lattice structure. Finally, this method is applied to the lightweight design of the lower limbs of a bionic quadruped robot, with experimental validation of its effectiveness. The research findings not only extend the scope of current lightweight design methods but also provide technical support and a data foundation for achieving the goals of high speed, precision, and lightweight in significant equipment development.

## Introduction

In recent years, the rapid development of major equipment fields such as aerospace vehicles, shipbuilding, and high-speed trains has increasingly emphasized the importance of high-load, lightweight structures^1,2^. Research on lightweight structural design plays a critical role in advancing manufacturing, enhancing the mechanical performance of mechanical components, and improving material utilization efficiency. Common lightweight structural forms employed in current technologies include thin-walled structures, honeycomb cores, lattice frameworks, and stiffened configurations^3^.

Lattice structures^4^, a lightweight structural form, were jointly proposed in 2000 by Professor Evana from Princeton University and Professor Hutchinson from Harvard University. The design approach involves three key steps: first, designing or selecting a type of lattice structure; second, filling the target design domain using methods such as arraying or adaptation; and finally, generating a design scheme with lattice characteristics^5^. Currently, lattice structure units are primarily categorized into three main types: bio-inspired structures^6^, structures based on the seven crystal systems^7^, and topologically optimized structures^8^.

The design of lattice structure units is often inspired by the exceptional structures and functions found in biological organisms^9,10^. San et al.^11^ investigated the performance differences in energy absorption among various bio-inspired lattice structures and provided a comprehensive summary of future research directions in this field. Xiang et al.^12^ conducted a biomimetic study on beetle skeletal structures and designed a tubular energy-absorbing structure resembling a honeycomb. By optimizing the distribution of the tubular lattice, they enhanced the energy absorption efficiency of the lattice structure.

Based on the characteristics of the seven crystal systems and quasi-lattices of alloy materials, lattice structure cell configurations have been developed. These include simple cubic (SC) cells^13^, body-centered cubic (BCC) cells^14^, face-centered cubic (FCC) cells^15^, tetrahedral cells (Tetrahedron)^16^, and octahedral cells (Octahedron)^17^. Li et al.^18^ calculated the equivalent elastic modulus of body-centered cubic (BCC) lattice units, analyzed the influence of lattice structural parameters on mechanical performance, and applied the design to mechanical components. Their findings demonstrated the significant advantages of lattice structures in lightweight design.

Topology-optimized^19^ lattice structure design is an emerging method in engineering, aiming to achieve structural lightweighting and performance optimization by refining the topology of the structure. Serdar et al.^20^ proposed a topology optimization algorithm to determine the optimal number of rings for architectural dome member groups. The algorithm can automatically generate the required data for the geometry of these domes, and its effectiveness and feasibility were validated through case studies.

In this article, innovative lattice structure unit configurations have been developed using topology optimization techniques, and a comprehensive library of these configurations has been created. The mechanical performance characteristics of various lattice units were systematically analyzed, leading to the proposal of a method for matching lattice unit configurations. This lightweight structural design has been successfully applied to the lower limbs of bionic robots.

## Performance characteristics and applications of the lattice structure unit

### Configuration design

Topology optimization^21^ thinking is to establish equivalent models of loads, constraints, etc. that are suitable for the actual working environment within a given design space, seeking the optimal allocation of materials.

This study abstracts tension-compression, bending, torsion, and their combined loading conditions from practical engineering scenarios. Using the Altair OptiStruct software and the variable-density method (SIMP), the optimal lattice unit cell topologies are derived under various loading conditions to minimize compliance.

Following the optimization design approach, the load and boundary conditions for the lattice structure unit are categorized into 20 distinct scenarios, as illustrated in Table 1.

**Table 1:**
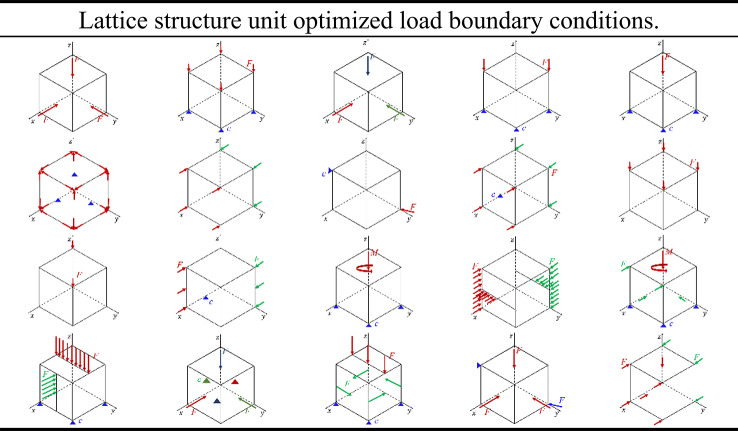
Lattice structure unit optimized load boundary conditions.

To optimize lattice unit configurations for components subjected to tension, compression, bending, torsion, and complex loading conditions, a library of 20 distinct lattice configurations has been established, as shown in Table 2. This study analyzes their mechanical behavior under compression, bending, and torsional loading, and explores their potential for achieving lightweight design goals.

Topological optimization significantly enhances structural performance and material utilization while fostering the development of novel lattice configurations. However, the lattice structures that emerge from this process often have intricate geometric features and irregular morphologies, complicating the manufacturing, analysis, and practical application of these structures. Thus, it is essential to regularize the optimized structures. Regularization facilitates the smoothing of sharp edges, the removal of overhanging features, and the mitigation of stress and strain concentrations, thereby enhancing the structural strength and stability.

**Table 2:**
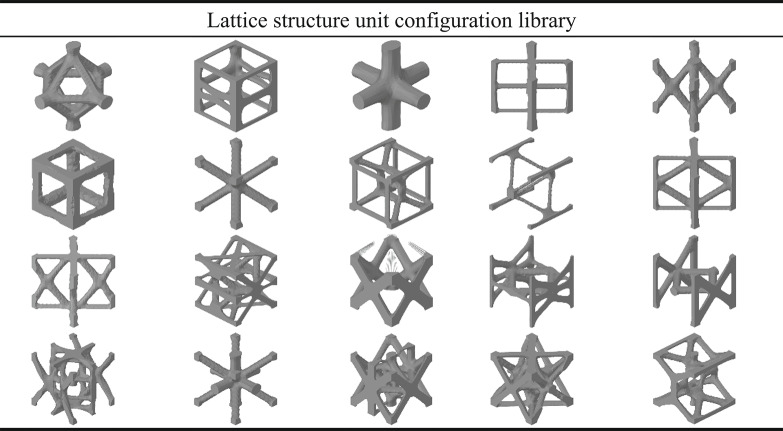
Optimization of load boundary conditions for lattice structure unit.

In this study, the structural regularization of the 20 designed lattice unit cells was performed using the 3D modeling software SolidWorks, where smoothing and geometric refinement were applied to enhance geometric uniformity and manufacturability. This process aims to improve manufacturability while maintaining adherence to design objectives and performance requirements. The configurations and scale parameters for these lattice units were defined based on the outcomes of topological optimization. Each of the 20 lattice types is then subjected to the regularization process. Furthermore, for units labeled *m*, *n*, and *o*, specific modifications were made based on their individual loading conditions and structural characteristics. These adjustments ensure that the units exhibit symmetrical and uniform geometrical features, as detailed in Table 3.

**Table 3:**
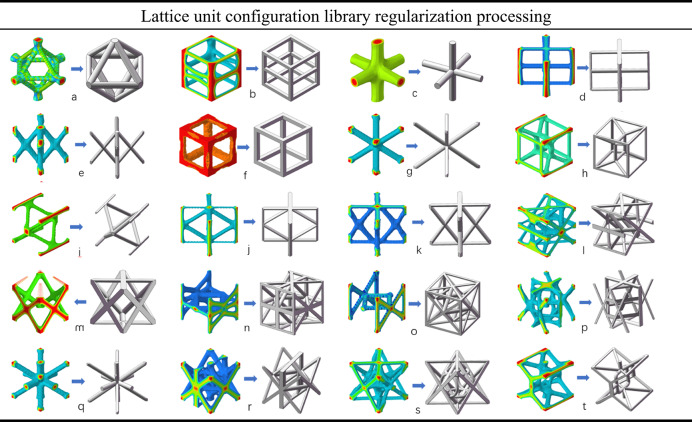
Regularization of the lattice structure unit configuration library.

### Evaluation standards for mechanical performance

To effectively utilize lattice structure units, a comprehensive and accurate assessment of their mechanical performance is necessary. The primary indicators for evaluating the load-bearing performance of these structures are compression, bending, and torsion.

Achieving lightweight design in lattice structures while meeting mechanical performance requirements necessitates a novel design approach. In this context, the concept of relative density for lattice structure units is introduced. Relative density^22^ refers to the ratio of the actual material volume within a lattice structure to the volume of a fully solid body occupying the same space. It is a dimensionless quantity typically ranging from 0 to 1. This metric is employed to compare the mechanical performance of the 20 different lattice unit cell configurations.

The density of lattice structural units is represented as follows:

1 $$\rho =\left( {V/{V_0}} \right){\rho _m}$$

The relative density of lattice structural units is represented as follows:

2 $${\rho _d}=\rho /{\rho _m}=V/{V_0}$$

In the formula: *p*_m_ is the density of the material, *kg/m*^*3*^; *V* is the volume of the unit cell porous structure, *mm*^*3*^; *V*_0_ is the total volume of the unit cell, *mm*^*3*^.

The results are as shown in Table 4.

**Table 4:**
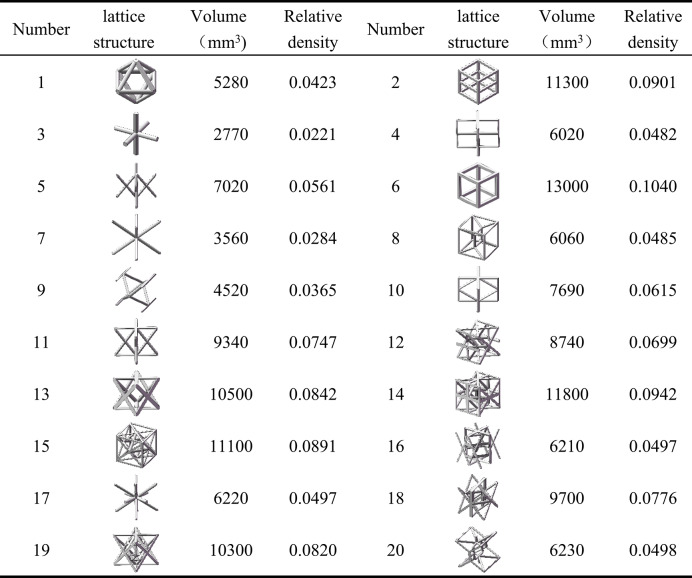
Relative density of lattice structural units.

Calculating the relative density of 20 structural units provides insights into how size parameters affect their performance. This analysis is crucial for further optimization. Lattice structural units must exhibit not only the trait of being lightweight but also possess excellent resistance to tension, compression, bending, and torsion. Therefore, it is imperative to assess the mechanical properties of lattice structures.

Based on the above two factors, this paper adopts specific stiffness^23^ as a comprehensive metric to evaluate both lightweight efficiency and mechanical performance. This approach facilitates a more in-depth investigation into the mechanical behavior of lattice structural units. The specific stiffness is a key indicator of a structure’s resistance to deformation, significantly influencing the load-bearing behavior of lattice structures. A higher specific stiffness can reduce structural deformation, preventing excessive deformation that leads to structural failure or performance degradation. By comparing the specific stiffness of various lattice units, the most appropriate lattice structure can be selected according to specific application requirements.

The specific mathematical formula is expressed as follows:

3 $$F=K \cdot \Delta L$$

In the formula, *F* is the external force on the object; *K* is the stiffness constant; Δ*L* is the deformation of the object.

In this paper, the relative density is used as a standardization tool to compare the stiffness performance of various structures more accurately.

4 $${T_s}=K/{\rho _d}$$

In the formula, *T*_*s*_ is the specific stiffness; *K* is the stiffness constant; *ρ*_*d*_ is the relative density of different structures.

The comparison of specific stiffness values reveals the strengths and limitations of structural performance at varying relative densities. A higher specific stiffness implies that the structure is less affected by external forces, while a lower stiffness indicates a more prone to deformation to such forces. This comparative analysis aids in comprehensive evaluation of the mechanical performance of different lattice structures. It provides a foundation for structural optimization in practical applications, guiding the development of designs that are both lightweight and capable of withstanding external forces.

### Mechanical performance

The conditions of compression, bending, and torsion can well simulate the main stress situations encountered by mechanical parts in practical work. This paper simplifies the analysis of the mechanical properties of lattice structural elements into compression, bending, and torsional situations.

1. Performance analysis of compression condition.

**Fig. 1:**
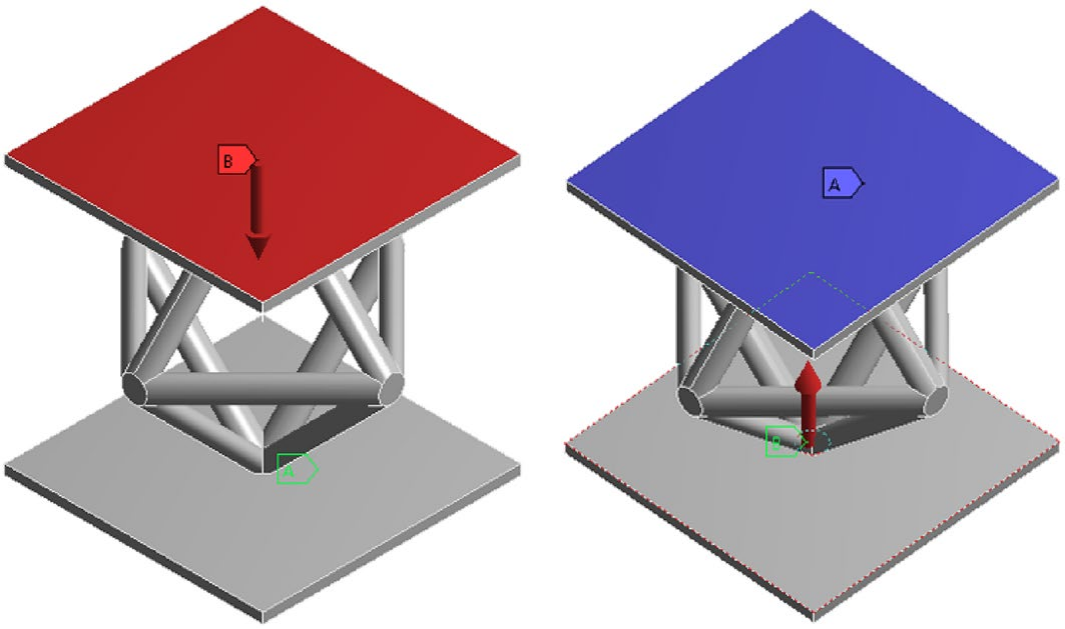
Compression condition.

The mechanical performance of the lattice structure unit is analyzed under compression conditions, and the upper and lower load-bearing boundary planes of the lattice structure are established, as shown in Fig. 1. In this study, the finite element model employs hexahedral 3D solid elements, meshed using a structured solid mesh. The model consists of approximately 32,000 elements and 35,301 nodes, with an element size of 5 mm × 5 mm × 5 mm to ensure sufficient resolution for the lattice core structure. To maintain consistency with subsequent experimental validation, the material used for analysis is 9400 photosensitive resin. The SIMP (Solid Isotropic Material with Penalization) density-based topology optimization method is adopted, with the objective of minimizing compliance under a volume fraction constraint. Static linear analysis is performed under quasi-static loading conditions.

**Fig. 2:**
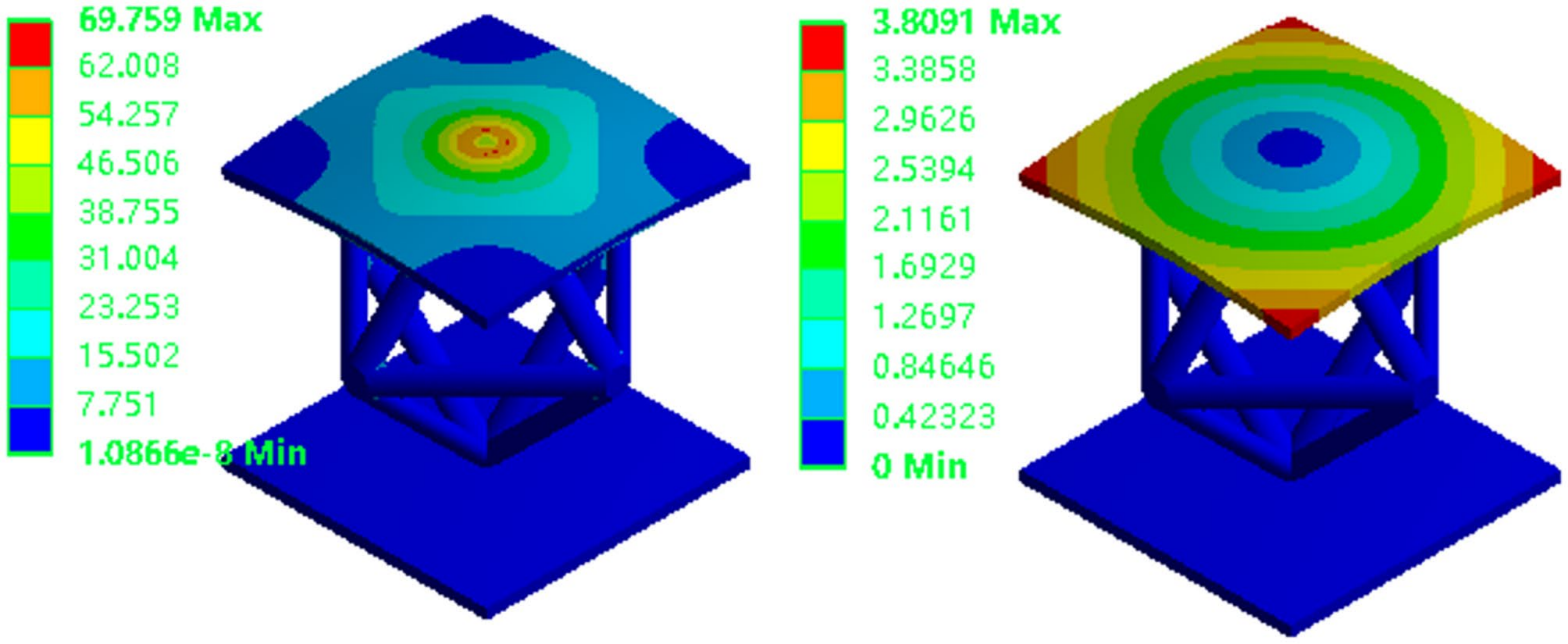
Compression equivalent stress and total deformation.

As shown in Fig. 2, the stress distribution across the members is symmetrical, reflecting the inherent symmetry of both the structure and the applied loads. Members located at the truss junctions and at the interfaces between the upper/lower panels and the core structure experience higher compressive stresses. This results in stress concentrations. The overall displacement direction of the structure aligns with that of the applied load. This observation is crucial for understanding how the structure responds to external forces and for identifying areas prone to stress concentration, which is essential for structural design and optimization.

(2) Performance analysis of bending conditions.

**Fig. 3:**
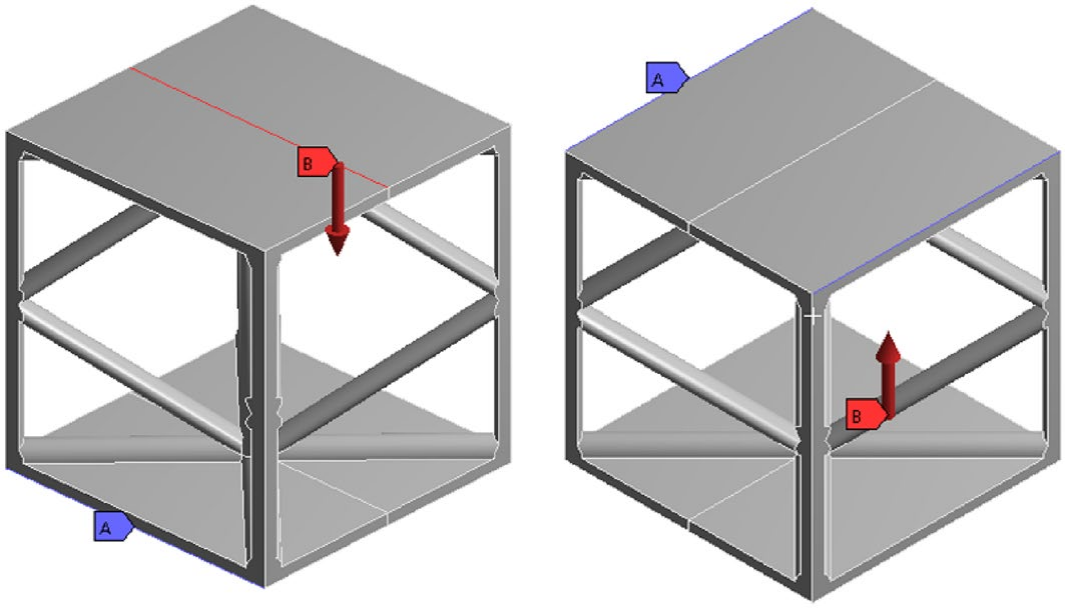
Bending condition.

The geometric model, material properties, and meshing are consistent with those used under compression conditions. As illustrated in Fig. 3, fully fixed boundary conditions are applied to the edges of the lower panel where the structure’s ends are in contact with the supports. A vertical line load is applied along the central axis of the upper panel on both sides. The load is applied progressively, increasing from 0 to 200 N.

Under three-point bending, the load is transmitted downward along the centerline of the upper panel. The primary load-bearing areas are located at the intersections of the upper panel’s centerline with the rod connections. The stress distribution is symmetrical along this centerline, consistent with the stress distribution patterns expected in three-point bending analysis.

**Fig. 4:**
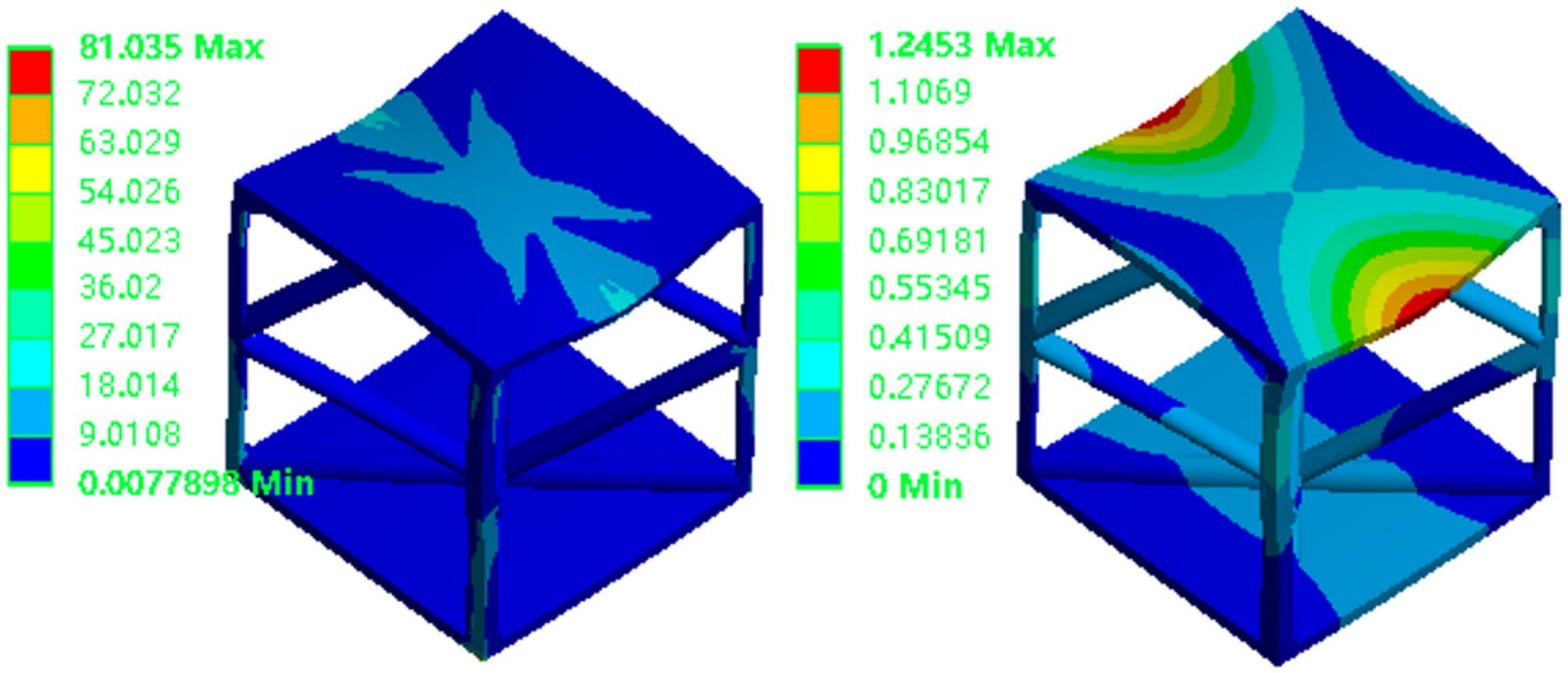
Bending equivalent stress and total deformation.

Figure 4 illustrates the displacement of the structure, which exhibits significant bending deformation. Both the nodal positions and the central region of the structure exhibit noticeable displacement. Significant displacements are observed in high-stress regions, particularly at joints and truss junctions. These regions indicate that the structure undergoes substantial deformation under loading. The observation of these displacements is crucial for assessing the structural integrity and performance under bending conditions.

(3) Mechanical performance analysis of torsional working conditions.

**Fig. 5:**
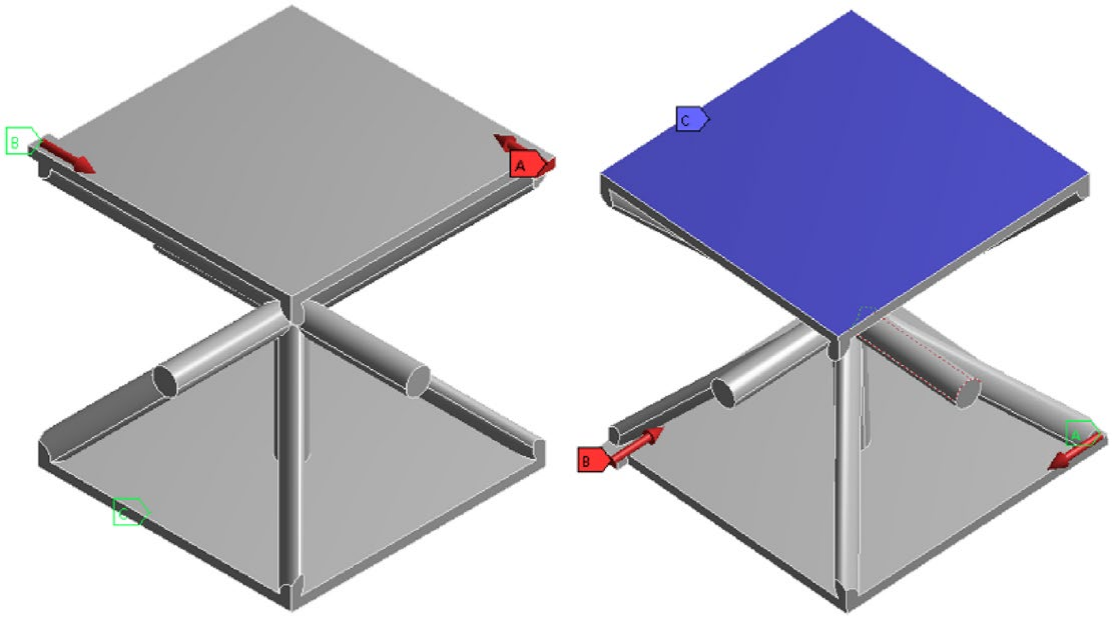
Torsional condition.

As depicted in Fig. 5, appropriate boundary and loading conditions are applied to the model to simulate torsional loading. Two equal-magnitude torques are applied to simulate the torsional effect. These torques, each with a magnitude of 50 N·m, are applied in opposite directions to the elevated end surfaces on either side of the model.

**Fig. 6:**
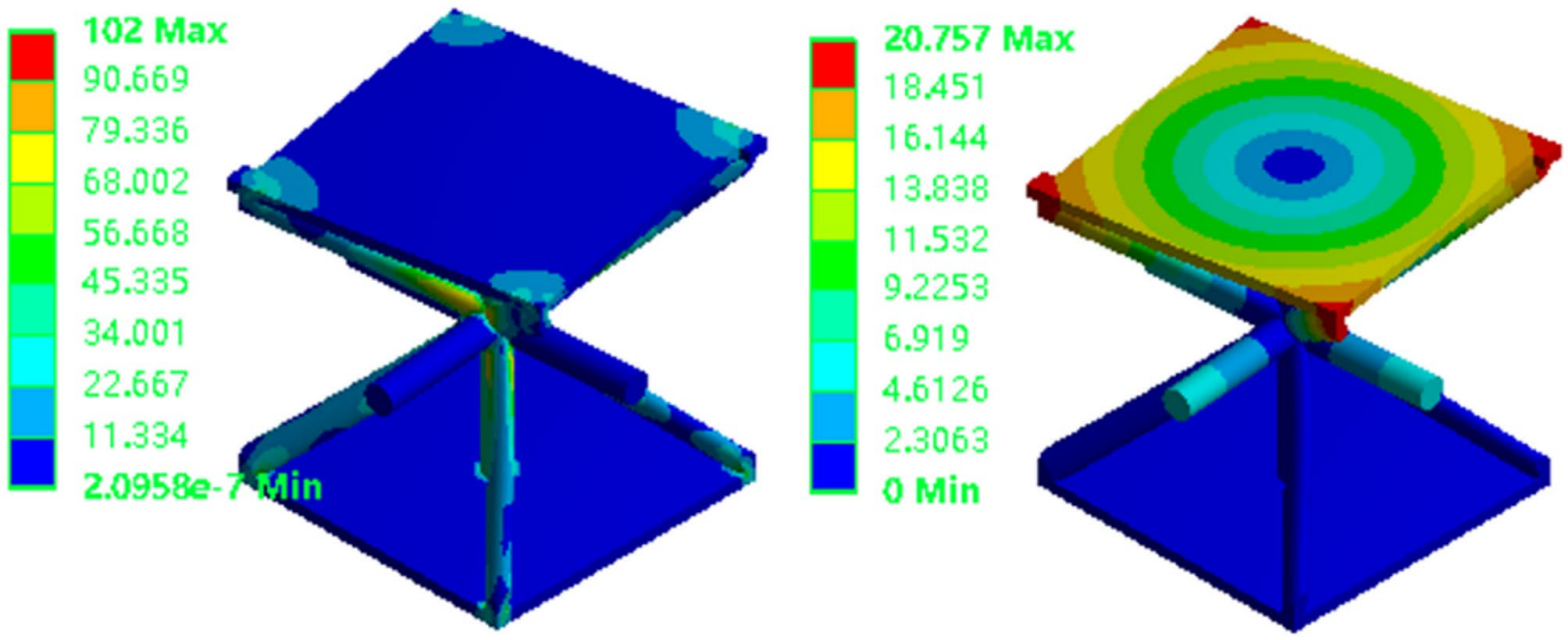
Torsional equivalent stress and total deformation.

Figure 6 illustrates the torsional stress and displacement within the structure. The applied torsional load induces rotational deformation, which manifests as torsional shear stress. The areas where stress concentration is most pronounced are the end face at the point of load application, the links of the lattice structure, and the joints where these links intersect. The maximum displacement occurs at the corner of the upper panel and gradually decreases toward the central region. This indicates a uniform pattern of torsional deformation across the structure. The lower panel, constrained by the boundaries and positioned far from the load application point, exhibits minimal displacement and deformation. These findings are critical for analyzing the structural response to torsional forces and for informing structural design to ensure optimal performance under such loading conditions.

### Mechanical behavior

**Fig. 7:**
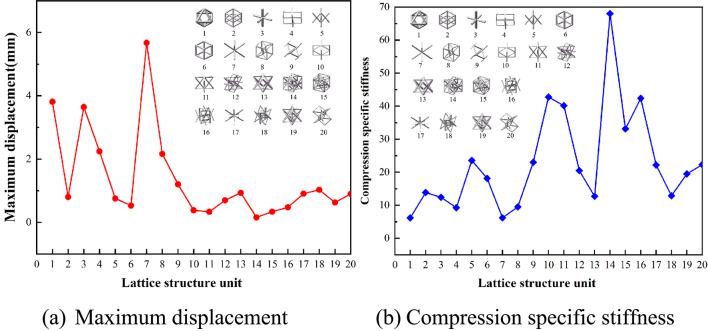
Compression mechanical behavior.

Based on the displacement data obtained from compression tests and the evaluation criteria described in Sect. 1.2, the mechanical performance under compression loading is assessed, as shown in Fig. 7. Lattice element No. 14 exhibits the highest specific stiffness, reaching a peak value of 65. Conversely, lattice element No. 7 demonstrates the lowest specific stiffness, with a value of only 6.20. This analysis enables a clear comparison of the mechanical performance of different lattice elements under compression, highlighting the importance of specific stiffness as a critical metric for structural evaluation.

**Fig. 8:**
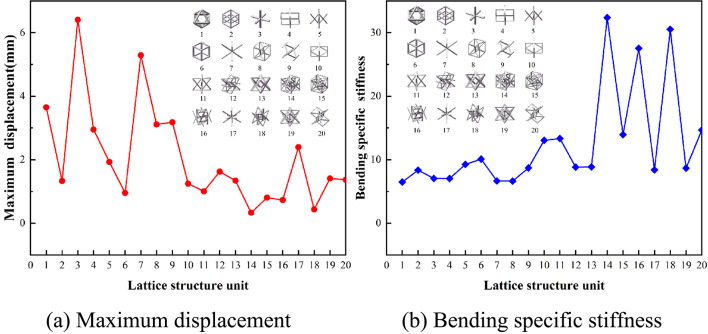
Bending mechanical behavior.

Figure 8 illustrates the bending response characteristics derived under bending conditions. Among the lattice structures tested, unit No. 14 demonstrates the highest stiffness at 32.38. In contrast, unit No. 1 exhibits the poorest bending performance, with a maximum displacement of 3.65 mm, a relative density of 0.0942, and the lowest specific stiffness at 6.48.

**Fig. 9:**
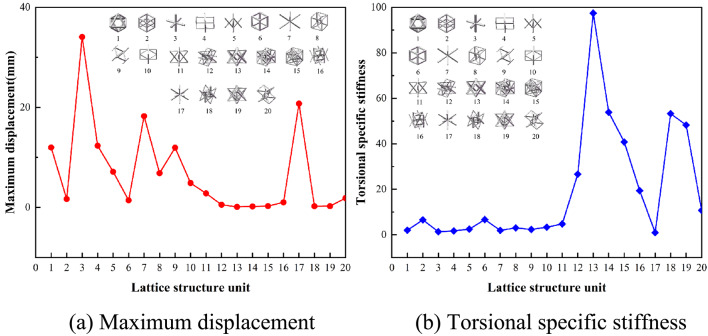
Torsional mechanical behavior.

Figure 9 depicts the mechanical behavior under torsional conditions. When subjected to torsion, the No. 14 lattice structure unit experiences a maximum displacement and deformation of 0.12 mm. With a relative density of 0.0842, it also exhibits the highest specific stiffness, at 97.44. In contrast, unit No. 17 exhibits the largest displacement under torsion, measuring 20.76 mm. With a relative density of 0.0497, it also has the lowest specific stiffness, recorded at 0.97.

In practical engineering applications, parts are rarely subjected to only a single condition such as compression, bending, or torsion. Instead, most parts operate under a variety of complex conditions, often under different types of loads simultaneously. Therefore, this paper integrates the various conditions that parts may be exposed to concurrently, as outlined in Table 5.

**Table 5:** Types of composite working conditions.

Number	Composite working condition
1	Compression & bending
2	Compression & torsional
3	Bending & torsional
4	Compression & bending & torsional

Under complex loading conditions, selecting an optimal lattice structure that satisfies lightweight design requirements requires a clear understanding of structural demands. Specifically, it is essential to evaluate the relative significance of compression, bending, and torsion, based on the types of applied loads, their directional distributions, and the functional requirements of the component. To address this, a weighting method is introduced to quantify the relative importance of each deformation mode. By assigning weights to the specific stiffness associated with each deformation mode—namely, in-plane compression, bending, and torsion—the method quantitatively represents the contribution of each mode to the overall loading scenario^24^. This weighted integration captures the influence of diverse loading conditions, thereby enhancing the relevance of the evaluation to real-world applications. As a result, it facilitates the selection of a lattice structure with optimal overall performance under multiple mechanical requirements.

The weight calculation method is

5 $${S_i}={W_1} \times {X_{i1}}+ \ldots +{W_n} \times {X_{in}}$$

In the formula, *X*_*in*_ indicates the value of *i* indicator in *n* samples or schemes; *W*_*n*_ represents the weight of each item; *n* indicates the number of data items.

These weights meet the following conditions:

6 $${W_1}+{W_2}+ \ldots +{W_n}=1$$

In the formula, 0 ≤ *W*_*i*_ ≤ 1, *i* takes 1, 2,…, *n*.

The mechanical loading characteristics of the parts were comprehensively analyzed under compound loading scenarios. The optimal structural scheme is selected by aligning the weight coefficient of the parts’ working conditions with the performance of the lattice structures. The weight coefficients for the four compound working conditions are presented in Table 6. These coefficients provide a quantitative assessment of the relative significance of each condition, guiding the selection of the most suitable lattice structure to meet the complex requirements of the application.

**Table 6:** Weight coefficient table of compound conditions.

Number	1		2		3		4		
Working condition	Compression	Bending	Compression	Torsional	Bending	Torsional	Compression	Bending	Torsional
1	0.49	0.51	0.76	0.24	0.77	0.23	0.42	0.44	0.13
2	0.62	0.38	0.68	0.32	0.56	0.44	0.48	0.29	0.23
3	0.64	0.36	0.90	0.10	0.84	0.16	0.60	0.34	0.06
4	0.57	0.43	0.85	0.15	0.81	0.19	0.52	0.39	0.09
5	0.72	0.28	0.90	0.10	0.79	0.21	0.67	0.26	0.07
6	0.64	0.36	0.73	0.27	0.60	0.40	0.52	0.29	0.19
7	0.48	0.52	0.76	0.24	0.78	0.22	0.42	0.45	0.13
8	0.59	0.41	0.76	0.24	0.69	0.31	0.50	0.35	0.16
9	0.73	0.27	0.91	0.09	0.79	0.21	0.68	0.26	0.07
10	0.77	0.23	0.93	0.07	0.80	0.20	0.72	0.22	0.06
11	0.75	0.25	0.89	0.11	0.74	0.26	0.69	0.23	0.08
12	0.70	0.30	0.43	0.57	0.25	0.75	0.37	0.16	0.48
13	0.59	0.41	0.12	0.88	0.08	0.92	0.11	0.07	0.82
14	0.68	0.32	0.56	0.44	0.38	0.62	0.44	0.21	0.35
15	0.70	0.30	0.45	0.55	0.25	0.75	0.38	0.16	0.46
16	0.61	0.39	0.69	0.31	0.59	0.41	0.47	0.31	0.22
17	0.73	0.27	0.96	0.04	0.90	0.10	0.70	0.27	0.03
18	0.30	0.70	0.19	0.81	0.36	0.64	0.13	0.32	0.55
19	0.69	0.31	0.29	0.71	0.15	0.85	0.26	0.11	0.63
20	0.60	0.40	0.68	0.32	0.58	0.42	0.47	0.31	0.22

**Fig. 10:**
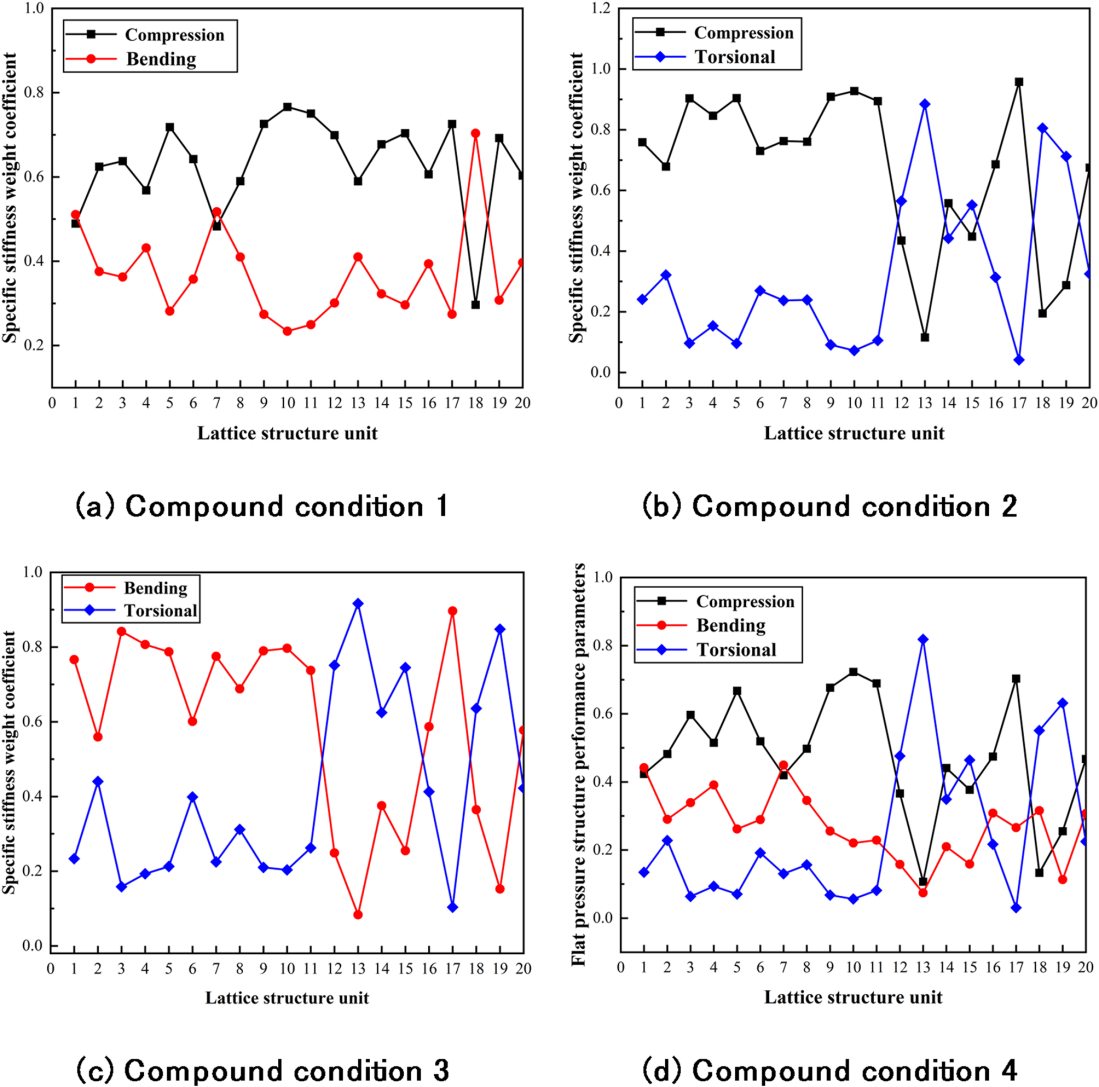
Mechanical performance of four compound conditions.

The mechanical performance of the four compound working conditions is shown in Fig. 10.

### Application method

To gain a more accurate understanding of the mechanical behavior of parts under complex working conditions, and to facilitate the selection of an appropriate lattice structural unit, the Analytic Hierarchy Process (AHP) is introduced in this section^25^. This method systematically analyzes the operational conditions of the parts and evaluates their mechanical performance under complex environments. Through the AHP, a structured and systematic approach is taken to evaluate and prioritize the various factors influencing the part’s performance. This analysis helps obtain a comprehensive understanding of the part’s behavior under specified conditions. It facilitates the informed selection of the lattice structural unit that best satisfies the design requirements.

Construct the decision matrix:

7 $$A=\left[ {\begin{array}{*{20}{c}} 1& \cdots &{{a_{1n}}} \\ \vdots & \ddots & \vdots \\ {{a_{n1}}}& \cdots &1 \end{array}} \right]$$

In the formula, *n* represents the number of working conditions; *a*_*ij*_ indicates how important factor *i* is relative to factor *j*.

The weight for each working condition corresponds to the principal eigenvector of the decision matrix. This eigenvector represents the relative importance of each condition. The resulting importance ratios for each condition are listed in Table 7. These ratios offer a clear view of the significance attributed to each working condition, providing a quantitative basis for decision-making in the context of the analysis.

**Table 7:** Importance ratio of the decision matrix.

Factor i and factor j	Quantized value
Equally important	1
Slightly important	3
Stronger Important	5
Strongly important	7
Extremely important	9
The middle of two adjacent judgments	2, 4, 6, 8,
Reciprocal	The ratio of *i* to *j* is the inverse of *j* to *i*

To prevent significant deviations in the eigenvalues and eigenvectors of the decision matrix that may arise from inconsistent judgments regarding the importance of each working condition, the Consistency Index (CI)^26^is utilized. This index assesses the level of consistency within the judgment matrix. The use of the Consistency Index ensures a reliable evaluation of the decision matrix, thereby enhancing the accuracy and reliability of the weight coefficients derived from it.

8 $$CI=\frac{{{\lambda _{\hbox{max} }} - n}}{{n - 1}}$$

In the formula, CI is the consistency index; *λ*_*max*_ is the maximum eigenvalue of the matrix. *n* is the order of the matrix.

When the consistency index CI = 0, the decision matrix is completely consistent. When the consistency index CI ≠ 0, the consistency ratio CR was introduced. When the order of the decision matrix is *n* > 2, if CR < 0.1, the consistency is acceptable. The expression of CR is

9 $$CR=\frac{{CI}}{{RI}}$$

In the formula, RI is the correction value.

Utilizing the aforementioned method, the working weight of the parts under complex conditions can be accurately calculated. This calculation is instrumental in matching the appropriate lattice structure unit.

## Experiments on the mechanical properties of lattice structural units

### Design of the experimental scheme of the lattice structure unit

All mechanical performance tests were conducted using an Instron 3369 dual-column universal testing machine, which has a maximum load capacity of 50 kN and a loading accuracy of ± 0.5 N. The experimental loading conditions included compression, bending, and torsion. Custom-designed fixtures were used to securely hold the specimens, ensuring consistent and uniform loading across all tests. Load–displacement data were collected and analyzed using the Bluehill software provided by Instron. Each lattice configuration was tested at least five times, and the average values were used for performance evaluation.

**Fig. 11:**
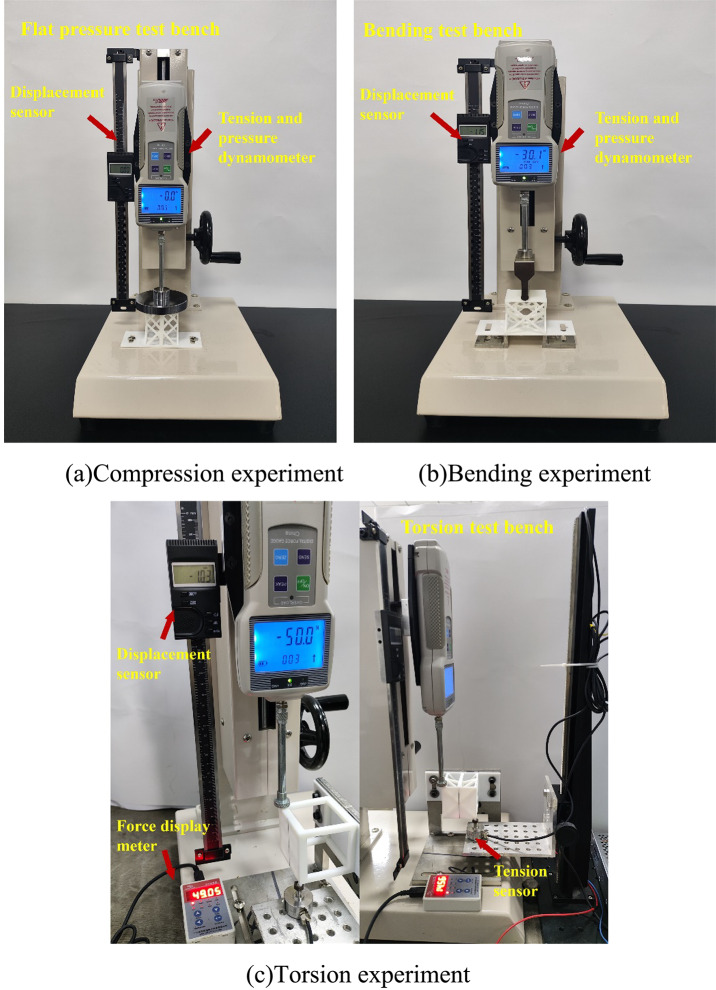
Specimen test bench.

For the compression test, as shown in Fig. 11(a), it is mainly composed of a compression test bench, a displacement sensor, and a tension-compression dynamometer. Pressure was applied by turning a side-mounted handwheel, while displacement and load values were monitored on the indicator. Loading was stopped once the applied force reached the predetermined target of 200 N. At that point, the final displacement and load values were recorded.

The bending test, shown in Fig. 11(b), utilized a bending test bench, a displacement sensor, and a tension-compression dynamometer. The loading plate was replaced with a three-point bending indenter, and a custom-designed bending fixture was used to secure the specimen. A vertical downward force was applied to induce bending deformation. Displacement changes were monitored in real time through the sensor. All other procedures followed the same protocol as the compression test, ensuring consistency and repeatability.

The torsion test setup, as depicted in Fig. 11(c), employed a torsion test bench, a displacement sensor, and a tension-compression dynamometer. The load is applied by turning the side displacement handwheel of the test bench and adjusting the height of the displacement slide bracket. This action causes the structure to undergo torsional deformation. Following the experimental requirements, the corresponding displacement response is recorded for each increment of 5 N of force. The force is gradually increased in a controlled manner until it reaches the target of 50 N.

During the experiment, the deformation and failure modes of various structures are systematically compared. Upon completing the test on one lattice structural unit, the structural specimen is replaced. This process is then repeated for the remaining 19 lattice structures. By systematically conducting and repeating the experiment for each unit, a comprehensive understanding of the performance characteristics of different lattice structures is achieved, laying a solid foundation for comparative analysis and subsequent research.

### Experimental performance and verification of lattice structural units

Compression, bending, and torsion tests were conducted on 20 distinct lattice structural units. For each experiment, the reaction force exerted by the indenter and the displacement data recorded by the sensor were extracted. Based on these datasets, load-displacement curves were generated for each lattice configuration. These curves provide a graphical representation of the structural response under different loading conditions, offering valuable insights into the mechanical behavior of each lattice unit.

**Fig. 12:**
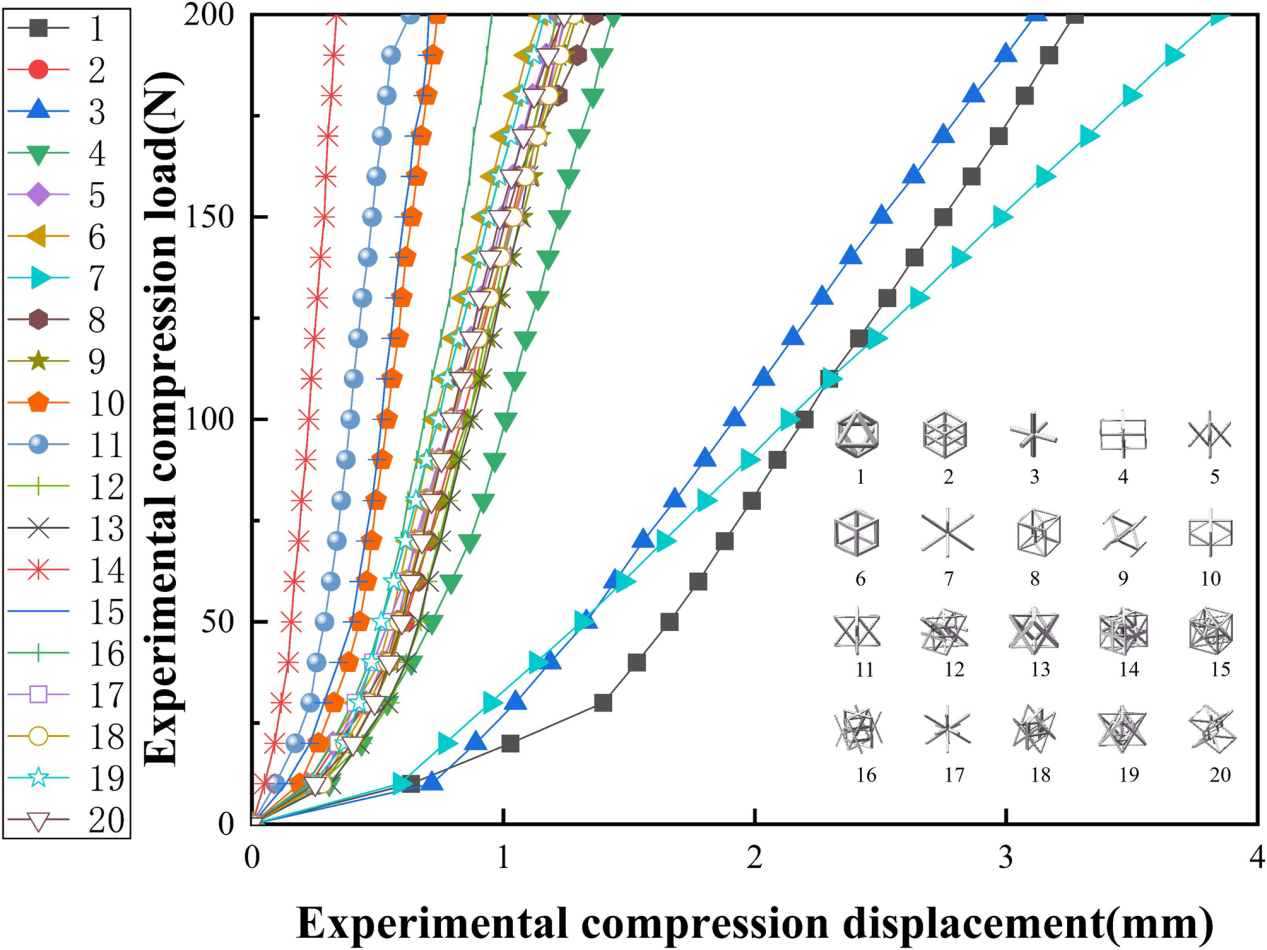
Compression experiment load displacement curve.

Figure 12 illustrates the load-displacement curve for the lattice structure specimens under the compression condition. Among all specimens tested under identical loading conditions, Specimen No. 7 exhibited the largest deformation, with a displacement of 3.84 mm, indicating the lowest stiffness. In contrast, Specimen No. 14 showed the smallest deformation, with a displacement of only 0.336 mm, suggesting a higher structural stiffness. This contrast highlights the significant variation in compressive performance across different lattice designs.

**Fig. 13:**
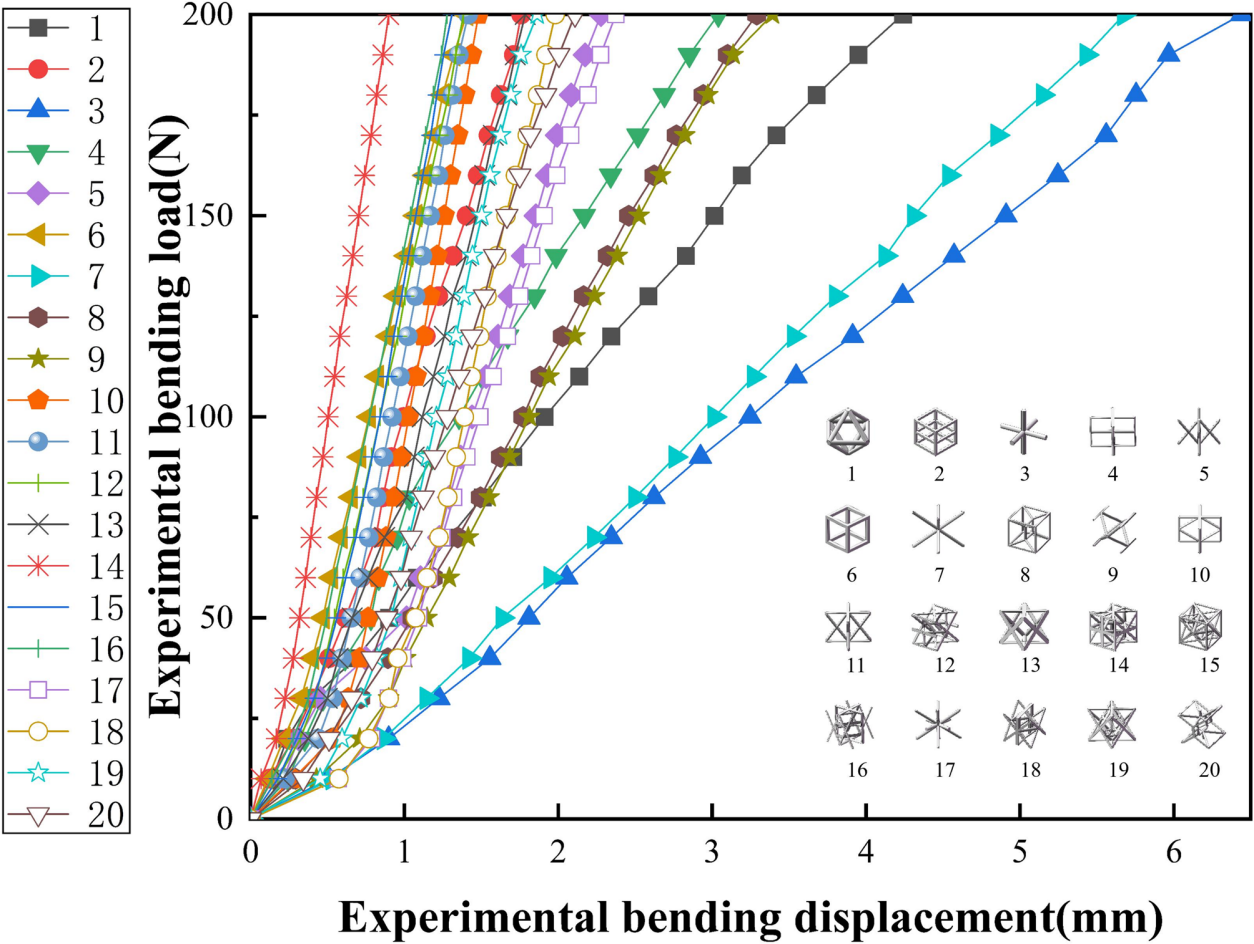
Bending experiment load displacement curve.

Figure 13 presents the load-displacement curves under three-point bending. A considerable difference in deformation behavior was observed among the specimens. Specimen No. 3 exhibited the greatest bending deformation, reaching 6.44 mm, while Specimen No. 16 experienced the least deformation, recording only 1.278 mm. These results reflect differences in flexural stiffness, with Specimen 3 being the most compliant and Specimen 16 the most rigid under the same bending load.

**Fig. 14:**
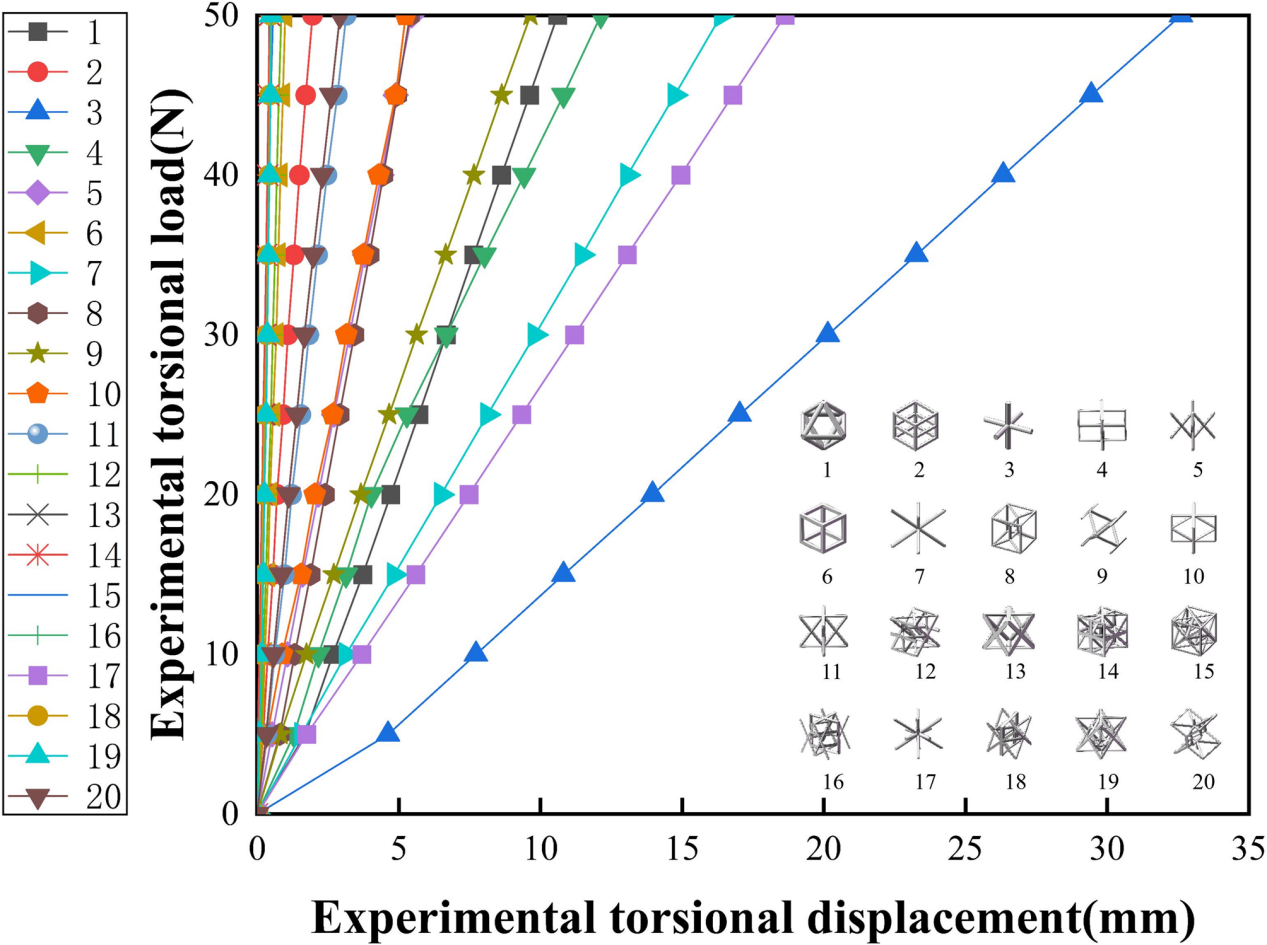
Torsion experiment load displacement curve.

Figure 14 presents the load-displacement curves of lattice structure units under torsional conditions. Under the same applied load, Specimen No. 3 exhibited the largest angular displacement, measuring 32.608 mm, whereas Specimen No. 13 showed the smallest, at 0.428 mm. These findings reveal a wide range of torsional stiffness characteristics, with Specimen 3 demonstrating higher compliance and Specimen 13 exhibiting greater resistance to torsional deformation.

**Fig. 15:**
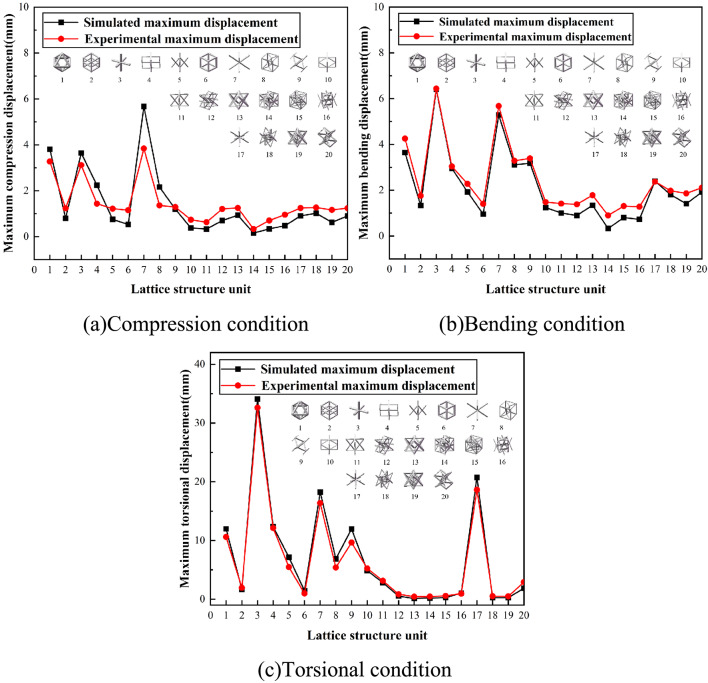
Comparison between simulation and experiment.

The experiments successfully validated the mechanical performance patterns of lattice structure units under compression, bending, and torsion. Figure 15 presents a comparison between the simulation and experimental results. The simulation results are in good agreement with the experimental outcomes, as shown in the figure. This comparison demonstrates the reliability of the simulation model in predicting lattice structure behavior under various loading conditions. It establishes a strong basis for further analysis and design optimization.

The relationship between displacement and load reveals the extent of structural deformation under varying applied forces, enabling assessment of structural stiffness. Experimental and simulation data complement each other and jointly contribute to the development and refinement of lightweight design.

## Lattice structure optimization design of robot lower limbs

### Force analysis of robot lower limbs

**Fig. 16:**
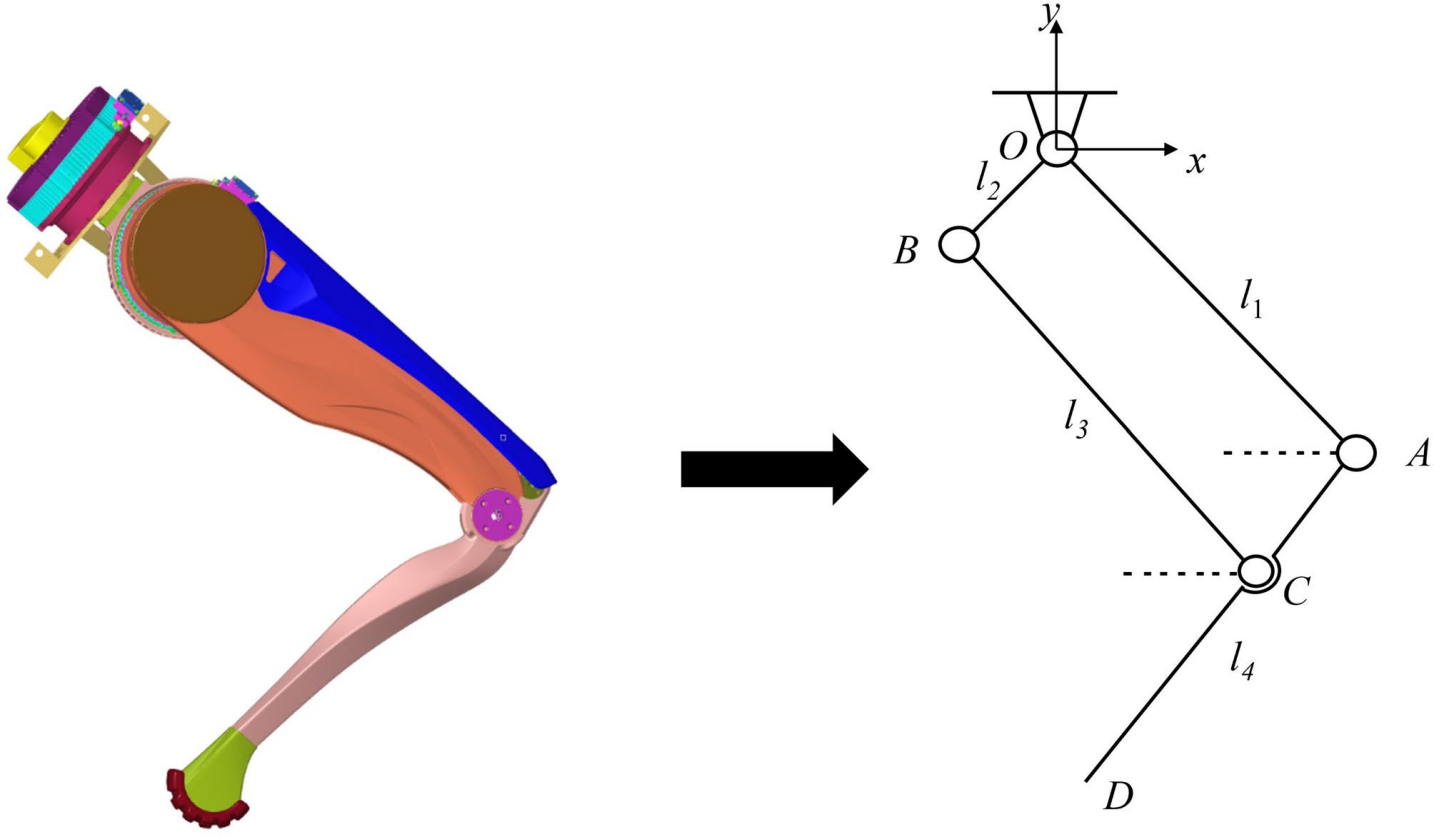
Schematic diagram of the leg mechanism of the bionic quadruped robot.

The bionic design of robot’s lower limbs is crucial for lightweight design. As shown in Fig. 16, a schematic diagram of the robot’s lower limb structure presents the components, connection methods, and the interrelationships among the parts. During the design process, particular attention is given to the mass of internal components housed within the torso and the structural redundancy of the shell. The robot’s upper body^27^, which weighs 20 kg, is designed to withstand a force of 200 N. The maximum joint torsional stress is calculated from a torsional moment of 10 N m using the motion model. Furthermore, to address dynamic gait forces such as those experienced during acceleration, the lower limbs are designed to withstand a bending load of 50 N.

### Finite element analysis and optimal design of robot lower limbs

**Fig. 17:**
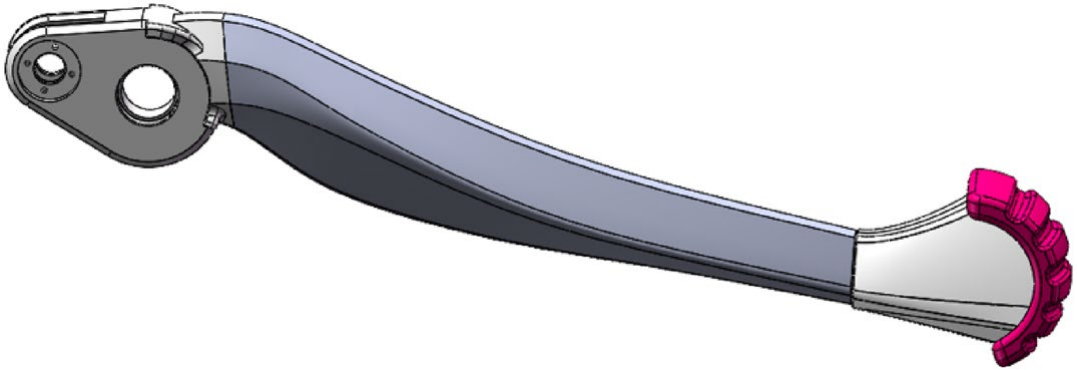
Schematic diagram of the leg mechanism of the bionic quadruped robot.

To improve computational efficiency, the three-dimensional model is simplified prior to analysis. Non-critical geometric features, such as holes and threads, are eliminated from the lower extremities. This simplification preserves the model’s essential mechanical characteristics while avoiding mesh distortion. Moreover, simplification improves mesh quality and bolsters the efficiency of the analysis. The refined is presented in Fig. 17, showcasing a balance between detail and performance that is essential for accurate simulation outcomes.

**Fig. 18:**
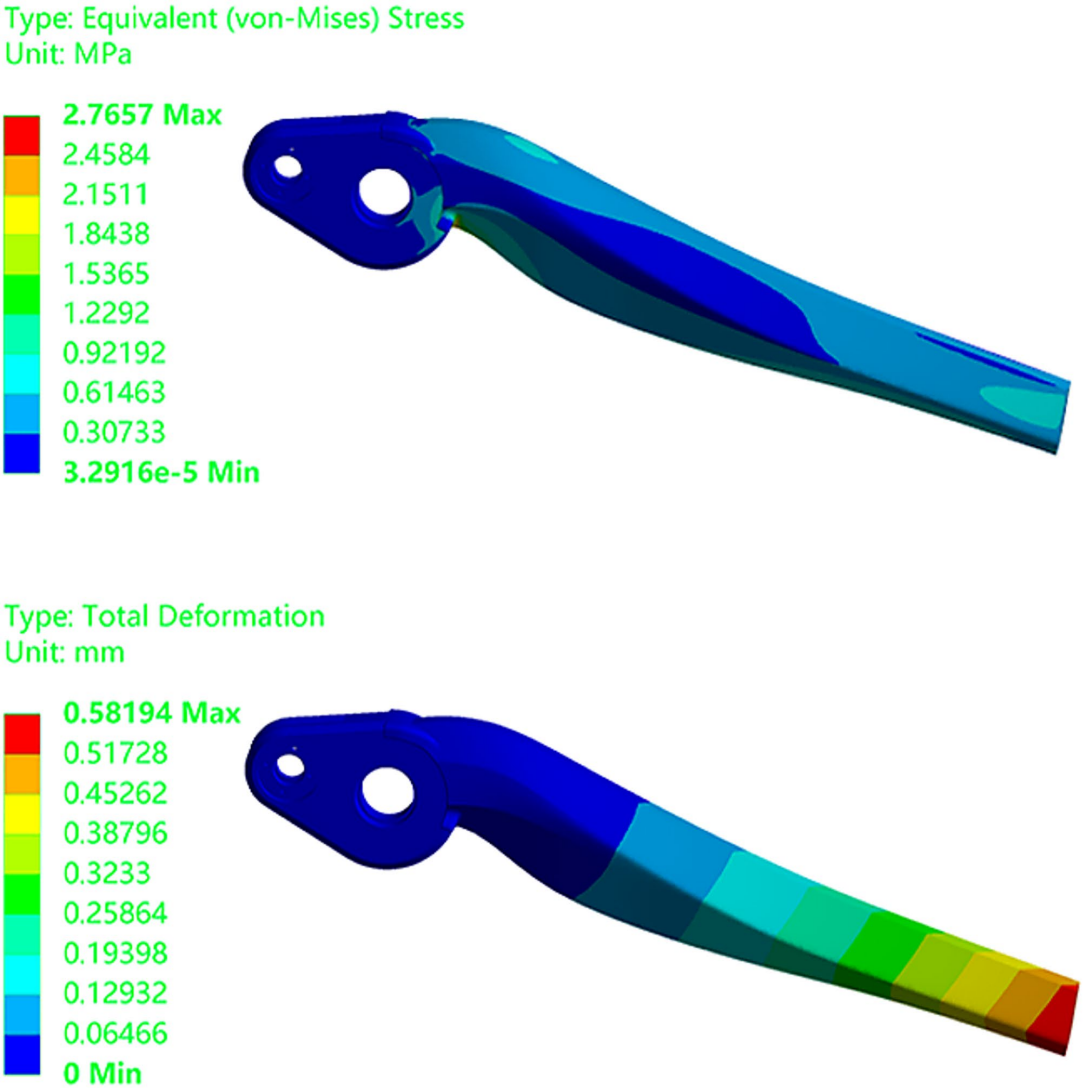
Leg structure equivalent stress and total deformation.

Figure 18 presents the stress and displacement contour plots of the leg structure. The simulation results indicate a maximum displacement of 0.58 mm, with stress concentrations primarily occurring at the joint connections. The maximum equivalent stress reaches 2.77 MPa, while stress levels in the central region of the structure are considerably lower. These stress values remain well below the material’s yield strength, indicating the potential for further weight reduction without compromising structural integrity. This insight is valuable for identifying regions where material can be safely removed, thereby supporting the overall lightweight design objectives.

**Fig. 19:**
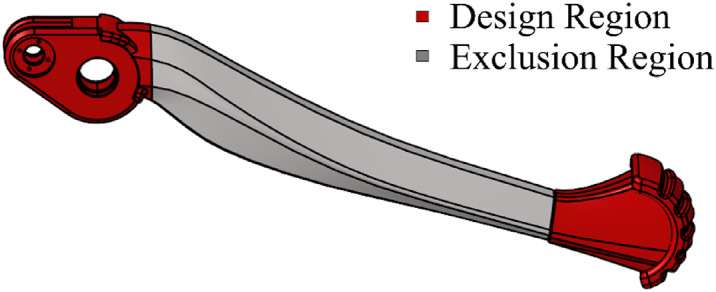
Leg structure optimization area settings.

Structural optimization targets the non-critical regions of the lower leg, while the essential components, such as the assembly holes, joints, soles, and connectors, remain intact. Based on the aforementioned conditions and additional design constraints, structural optimization is performed on the lower leg. As depicted in Fig. 19, the calf region is segmented into design and non-design domains. This strategic approach ensures that the optimization enhances the efficiency of the structure without altering the critical functional elements.

**Fig. 20:**
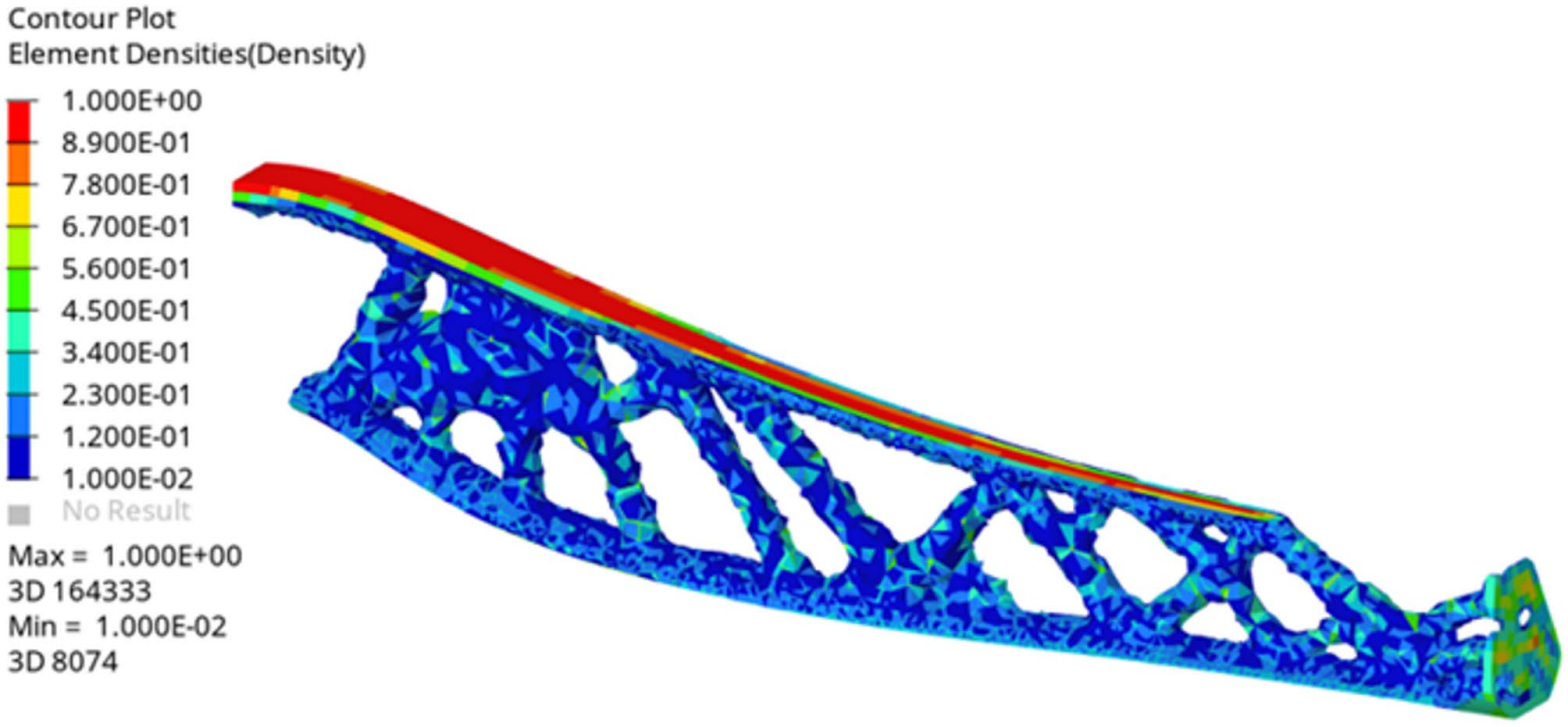
Optimized leg structure.

For the topology optimization of the connecting rod, symmetry constraints are applied, and a variable density method is utilized. This approach incorporates both strain energy minimization and volume fraction constraints to iteratively refine the structure. The optimization results are displayed in Fig. 20, demonstrating the optimal material distribution achieved through this systematic process. Material is effectively redistributed to maximize mechanical performance while minimizing weight, achieving a well-balanced design that satisfies both functional and structural efficiency requirements.

### Lattice structure filling and performance analysis

Considering the operating environment and functional characteristics of the bionic robot, the relative importance of the three loading conditions—compression, bending, and torsion—was evaluated using the lattice structure application method described in Sect. 1.5. The pressure exerted by the mechanism significantly outweighs the torque due to deflection; thus, it is considered of high importance, with a weighting factor of 7. Compared to torsion, pressure is considered more critical than the dynamic bending response. Therefore, it is assigned a weighting factor of 5. In practical terms, the comparison between torsion and bending is regarded as moderately important; hence, it is given a weighting factor of 3.

The decision matrix is thus constructed as follows:

10 $$A=\left[ {\begin{array}{*{20}{c}} 1&5&7 \\ {\frac{1}{5}}&1&3 \\ {\frac{1}{7}}&{\frac{1}{3}}&1 \end{array}} \right]$$

Using MATLAB, the eigenvalue problem was solved, yielding a maximum eigenvalue of 3.0649. Substituting this value yields a Consistency Index (CI) of 0.3245. Based on the consistency criterion, the Consistency Ratio (CR) is calculated to be 0.056, indicating that the decision matrix is fully consistent. The eigenvector corresponding to the matrix’s maximum eigenvalue is (0.9628, 0.2483, 0.1097)^T^. After normalization, the weight coefficients for the lower extremity structure of the bionic robot under complex conditions are determined to be 0.73, 0.19, and 0.08, respectively. These coefficients enable the assessment of the mechanical properties of the bionic robot’s lower limb structure. They provide a clear basis for designing and optimizing the robot’s structural components to meet the demands of complex operational conditions.

**Fig. 21:**
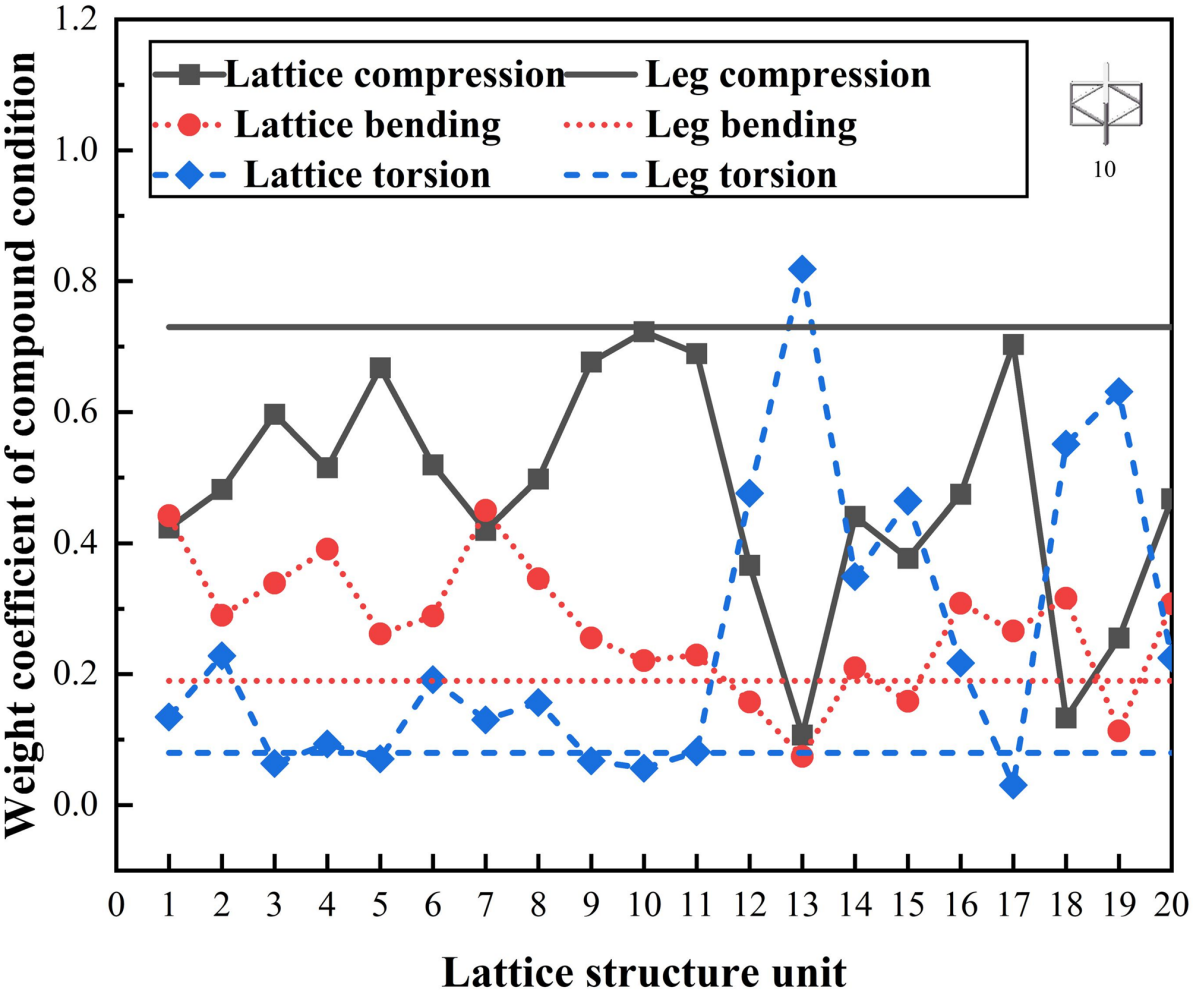
Matching diagram of lattice structure.

The matching diagram of the lattice structure is presented in Fig. 21. By assessing the mechanical properties of the lattice structure unit together with those of the bionic robot’s lower limb structure, the 10th lattice structure unit was ultimately selected for the filling process. This selection ensures that the chosen lattice structure will effectively meet the performance requirements of the robot’s lower limb, providing a balance between structural integrity and the desired lightweight design.

**Fig. 22:**
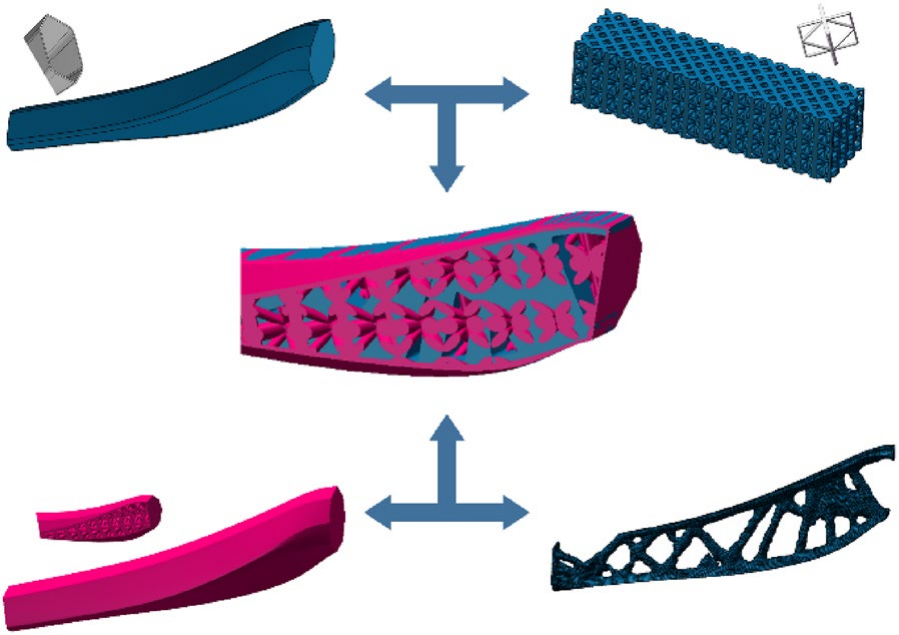
Lattice structure filling the leg structure design.

**Fig. 23:**
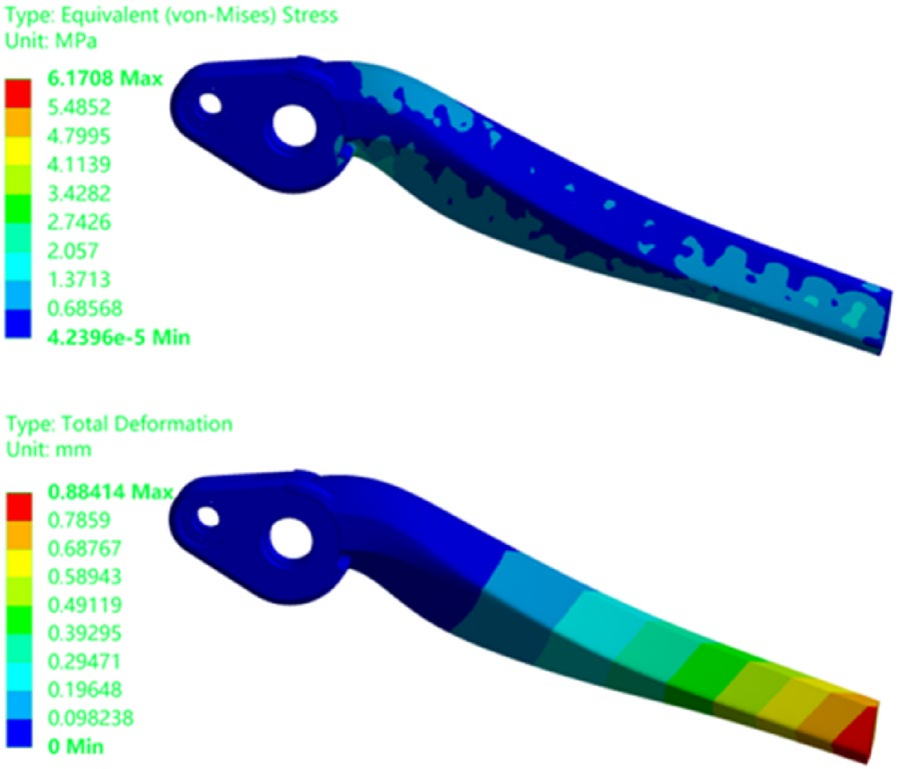
Optimization filling structure equivalent stress and total deformation.

Through design and filling processes, the final optimized structure was obtained, as depicted in Fig. 22. Using the optimized model and the finite element boundary conditions for the solid leg structure, the optimal filling model was simulated. The simulation produced stress and displacement contour maps, as shown in Fig. 23.

As detailed in Table 8, following optimization, the leg structure’s volume has been reduced by 23.03%, and its stiffness has increased by a factor of 1.97. Correspondingly, the overall weight decreased by over 23%. The lightweight methodologies, including topology optimization and lattice structures, have been successfully integrated. The objective of achieving a lightweight lower limb structure for the bionic robot has been accomplished. Furthermore, the superior performance potential of the lattice structure unit has been effectively harnessed, demonstrating the efficacy of these advanced design techniques in creating efficient and lightweight robotic components.

**Table 8:** Leg optimization filling data of bionic robot.

Model	Weight (kg)	Volume (mm^3^)	Maximum displacement (mm)	Maximum stress (MPa)
Initial structure	0.36	323,250	0.58	2.76
Optimized structure	0.29	248,820	0.89	6.17

## Experimental design and research of robot lower limbs

### Experiment construction and design

In this section, the design of pertinent experiments is conducted to evaluate the mechanical performance of the optimized bionic robot lower limb filled with lattice unit structures under various loading conditions. These experiments provided a comprehensive assessment of the lower limb’s performance across different scenarios, ensuring that the design meets the required specifications and is capable of withstanding the expected loads and stresses.

**Fig. 24:**
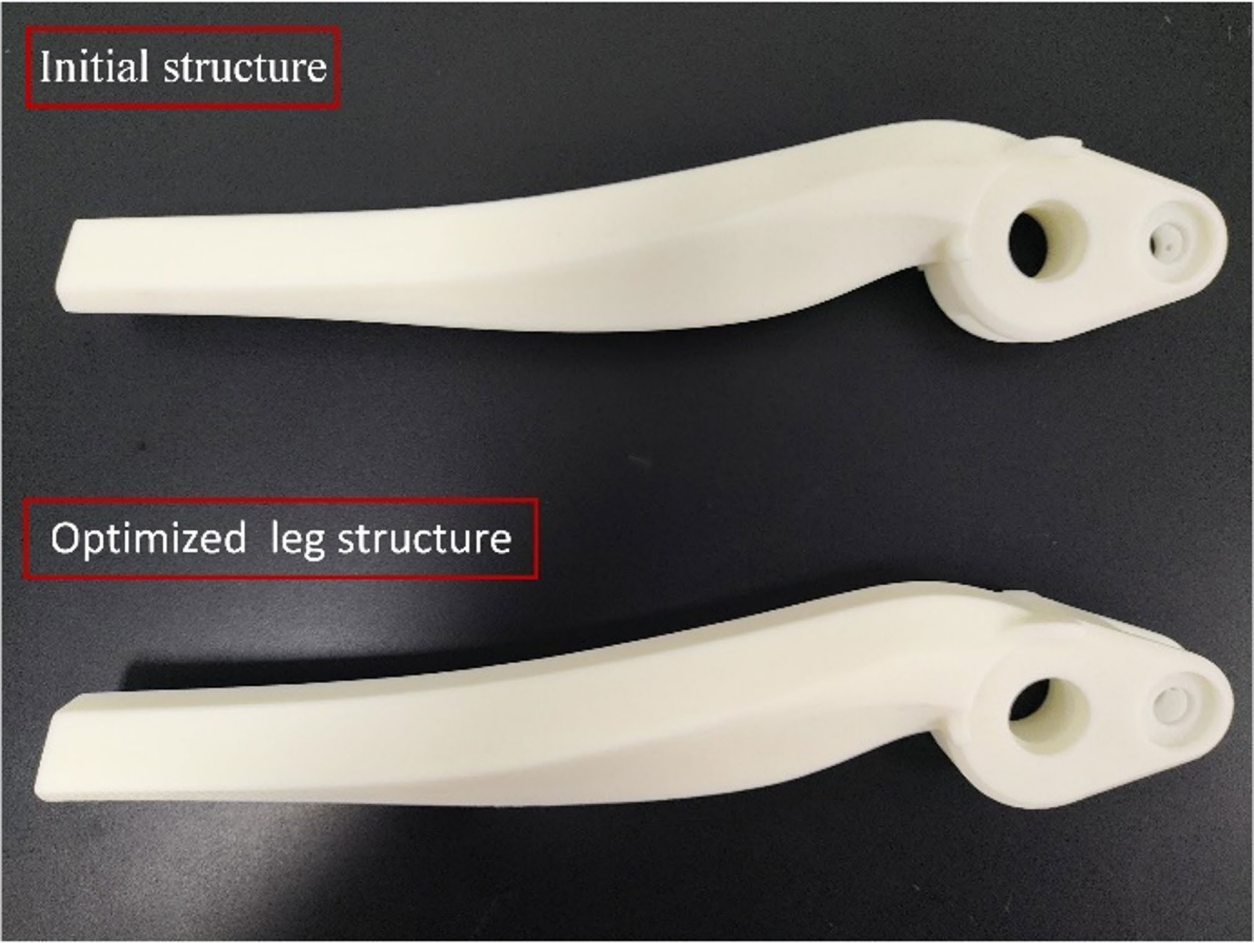
Printed model of leg structure.

The leg structure of the bionic quadruped robot was fabricated using Stereolithography Apparatus (SLA) technology. This advanced manufacturing technique enables precise realization of the intricate geometries of the lattice structure. The outcome of this process, as depicted in Fig. 24, serves to validate the practical feasibility of the design for the lattice-structured leg, which is based on the principles of topological optimization.

**Fig. 25:**
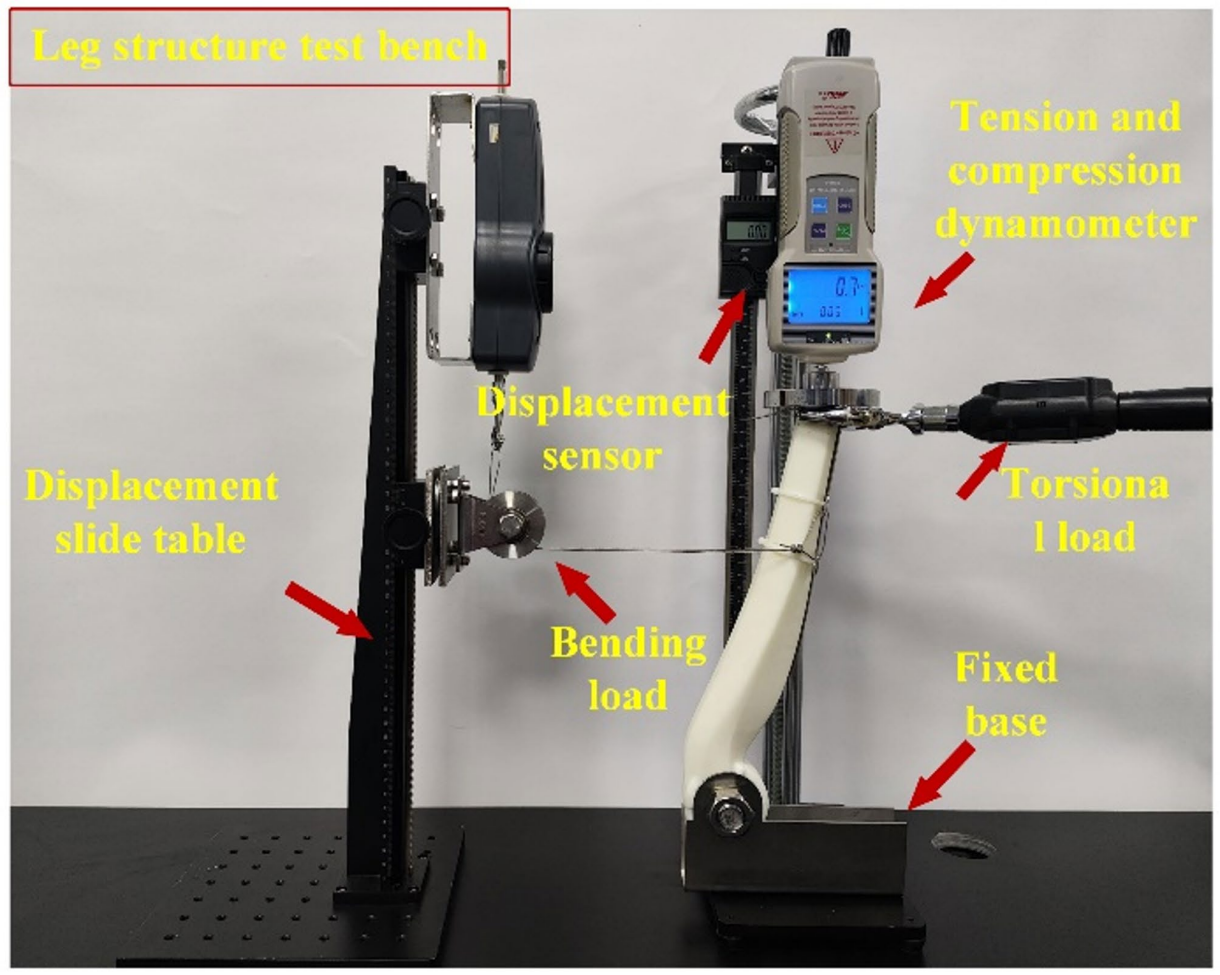
Lower limb structure experimental bench of the bionic robot.

The purpose of this experiment is to assess the mechanical properties of the bionic robot’s lower limb structure, both before and after optimization. The goal is to compare the mechanical strength of two different configurations of the component under identical complex working conditions. In accordance with the mechanical testing requirements, a specific test platform was constructed, as illustrated in Fig. 25. This platform is tailored to accurately measure and evaluate the performance of the robot’s lower limbs under the specified conditions.

By the testing requirements, static mechanical tests under multiple conditions were conducted. Data are recorded and analyzed to evaluate the performance. The same working conditions are applied to two different leg structures to compare their mechanical performance. This method effectively validates the feasibility of the lightweight design approach using lattice structure filling. The experiment confirms whether the structural design meets the required mechanical performance standards after optimization.

### Experimental results and verification

**Fig. 26:**
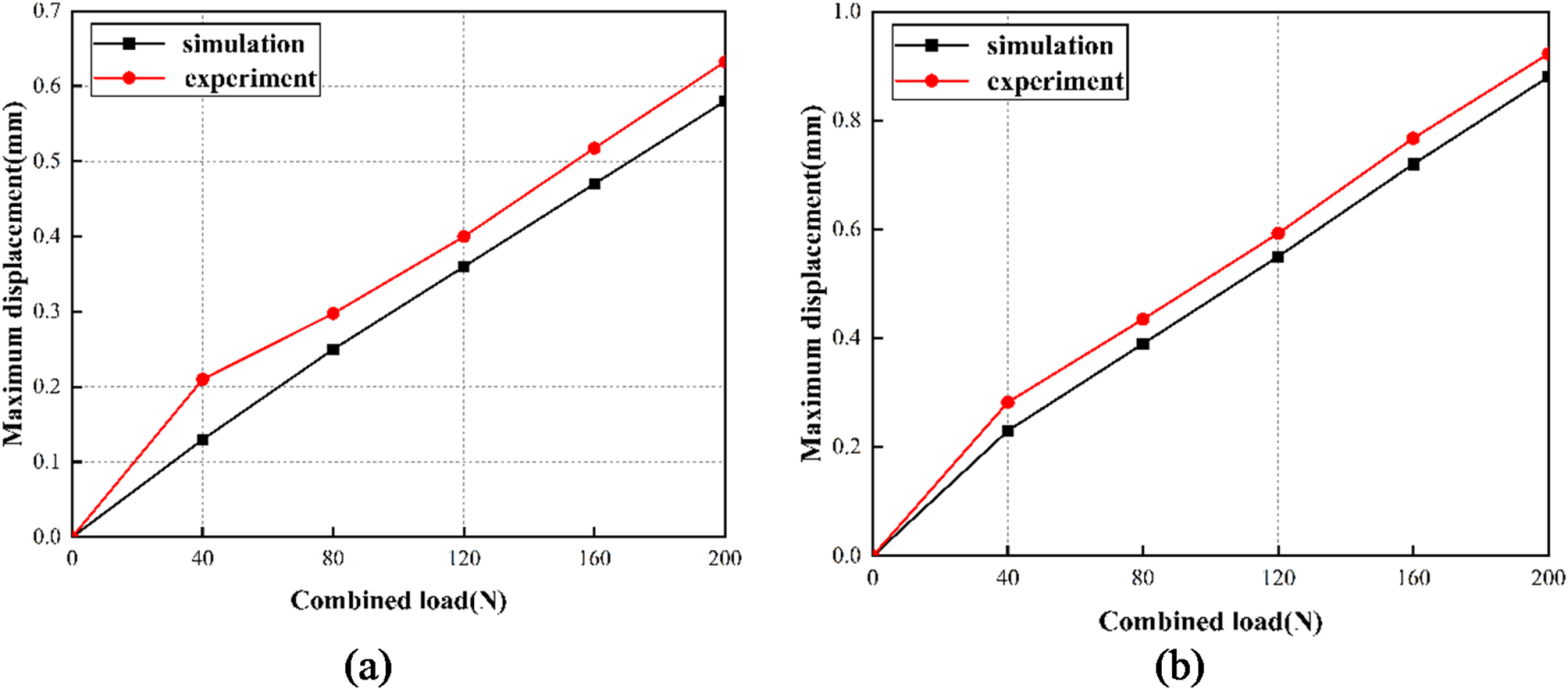
Comparison of experimental results. **(a)** Initial leg structure simulation and experimental results. **(b)** Optimized leg structure simulation and experimental results.

The data from the two sets of experimental tests were meticulously extracted and analyzed. The compiled data were then charted as a load-displacement curve, as depicted in Fig. 26. The results show that displacement progressively increases with the increasing load. Comparison of the experimental and simulation results for the two structures confirms the accuracy and reliability of the lightweight optimization of the bionic robot’s lower limb structure.

This outcome offers a novel perspective for the design of bionic robots. Concurrently, it delivers valuable insights for other domains engaged in the optimization and lightweight design of complex three-dimensional structures. The innovative approach and findings from this research have broad implications, potentially influencing and enhancing design practices across various engineering disciplines.

## Conclusion

This paper presents an innovative library of lattice structures, developed utilizing topological optimization. The mechanical performance of these structures is rigorously analyzed and verified through theoretical analysis and experimental testing. Furthermore, the paper explores the application of lightweight design through lattice structure filling in the context of bionic robot lower limb research.

1. An innovative configuration library of lattice structure units is constructed using topological optimization technology. This library characterizes the mechanical properties of the lattice units. The comprehensive evaluation methods and simulation analyses provide a valuable reference for understanding the mechanical behavior of these lattice units.
2. A comprehensive experimental protocol including compression, bending, and torsion tests was meticulously designed. These experiments rigorously evaluated the mechanical properties of the lattice elements, successfully verifying their structural integrity and performance.
3. The mechanical characteristics of the lattice structure unit were matched to those of the robot’s lower limb structure. Through this matching process, the optimal lattice structure unit is identified and selected to accomplish the lightweight design. The results demonstrate that the optimized lower limb structure of the bionic robot, both before and after the lattice infill, meets the application requirements. The specific stiffness of the entire structure has been enhanced by a factor of 1.97.
4. The mechanical properties of the lattice units were rigorously tested, and verification experiments validated the design of the bionic robot leg. This process confirmed the reliability and effectiveness of the optimal design scheme for the leg mechanism of the bionic quadruped robot.

## Data Availability

The datasets used and/or analysed during the current study available from the corresponding author on reasonable request.
